# Co-occurring KRAS mutation/LKB1 loss in non-small cell lung cancer cells results in enhanced metabolic activity susceptible to caloric restriction: an in vitro integrated multilevel approach

**DOI:** 10.1186/s13046-018-0954-5

**Published:** 2018-12-04

**Authors:** Elisa Caiola, Francesca Falcetta, Silvia Giordano, Mirko Marabese, Marina C. Garassino, Massimo Broggini, Roberta Pastorelli, Laura Brunelli

**Affiliations:** 1 Laboratory of Mass Spectrometry, Department of Environmental Health Sciences, Istituto di Ricerche Farmacologiche Mario Negri IRCCS, 20156 Milan, Italy; 2 Laboratory of Molecular Pharmacology, Department of Oncology, Istituto di Ricerche Farmacologiche Mario Negri IRCCS, Milan, Italy; 3 Laboratory of Cancer Pharmacology, Department of Oncology, Istituto di Ricerche Farmacologiche Mario Negri IRCCS, Milan, Italy; 4 Thoracic Oncology, Department of Medical Oncology, Fondazione IRCCS Istituto Nazionale dei Tumori, Milan, Italy

**Keywords:** NSCLC, KRAS^G12C^ mutation, LKB1 loss, Co-occurring genetic lesions, Metabolomics, Caloric restriction

## Abstract

**Background:**

Non–small-cell lung cancer (NSCLC) is a heterogeneous disease, with multiple different oncogenic mutations. Approximately 25–30% of NSCLC patients present KRAS mutations, which confer poor prognosis and high risk of tumor recurrence. About half of NSCLCs with activating KRAS lesions also have deletions or inactivating mutations in the serine/threonine kinase 11 (LKB1) gene. Loss of LKB1 on a KRAS-mutant background may represent a significant source of heterogeneity contributing to poor response to therapy.

**Methods:**

Here, we employed an integrated multilevel proteomics, metabolomics and functional in-vitro approach in NSCLC H1299 isogenic cells to define their metabolic state associated with the presence of different genetic background. Protein levels were obtained by label free and single reaction monitoring (SRM)-based proteomics. The metabolic state was studied coupling targeted and untargeted mass spectrometry (MS) strategy. In vitro metabolic dependencies were evaluated using 2-deoxy glucose (2-DG) treatment or glucose/glutamine nutrient limitation.

**Results:**

Here we demonstrate that co-occurring KRAS mutation/LKB1 loss in NSCLC cells allowed efficient exploitation of glycolysis and oxidative phosphorylation, when compared to cells with each single oncologic genotype. The enhanced metabolic activity rendered the viability of cells with both genetic lesions susceptible towards nutrient limitation.

**Conclusions:**

Co-occurrence of KRAS mutation and LKB1 loss in NSCLC cells induced an enhanced metabolic activity mirrored by a growth rate vulnerability under limited nutrient conditions relative to cells with the single oncogenetic lesions. Our results hint at the possibility that energy stress induced by calorie restriction regimens may sensitize NSCLCs with these co-occurring lesions to cytotoxic chemotherapy.

**Electronic supplementary material:**

The online version of this article (10.1186/s13046-018-0954-5) contains supplementary material, which is available to authorized users.

## Background

Non–small cell lung cancer (NSCLC) is a heterogeneous disease, with multiple different oncogenic driver mutations representing potential therapeutic targets [1–3]. Approximately 25% of NSCLC patients present *KRAS* mutations, which confer poor prognosis and high risk of disease recurrence [4, 5]. Currently, there are no successful treatment strategies that target KRAS mutant tumors [6–8]. Oncogenic KRAS has been shown to be a key factor in promoting metabolic rewiring, although the specific metabolic actors may differ depending on tumour type and genetic context [9–12]. In NSCLC, abnormal activation of KRAS enhances glucose metabolism to fuel oxidative phosphorylation and increases glutamine metabolism, the latter feeding mitochondria and maintaining the redox balance through glutathione biosynthesis [13–16].

Approximately half of NSCLC patients with activating *KRAS* lesions have also deletions or inactivating mutations in the serine/threonine kinase 11 gene (*LKB1/STK11*) [17–20]. Alterations in LKB1 are spread all along the gene and comprise deletions, insertions, frameshift, nonsense and missense mutations. As recently reported, *STK11/LKB1* mutations were in their overwhelming majority predicted to be deleterious for protein function [20]. LKB1 is a tumor suppressor that phosphorylates and activates several downstream targets to regulate signal transduction, energy sensing and cell polarity [21, 22]. It has a pivotal role in metabolic reprogramming and nutrient sensing, mainly through its ability to activate AMP-activated protein kinase (AMPK) [19, 23–26]. Inactivated *LKB1* is found in a wide range of human cancers including those of the pancreas, cervix and lung [27, 28].

The role of *KRAS* mutations and their potential association with other common genetic lung cancer lesions (*LKB1*, *TP53*) has recently been investigated in different cohorts of human lung adenocarcinomas using transcriptional, mutational, copy-number and proteomic data. These studies highlighted how *LKB1* inactivation is significantly associated with *KRAS* mutations compared to *TP53* deletion and that co-occurrence of *KRAS* mutation with inactivation of *LKB1*, *TP53* or *CDKN2A/B* genes generates different tumor subsets with distinct biology, immune profiles, and therapeutic vulnerabilities [29]. The co-occurrence of *KRAS* mutation and *LKB1* loss has been demonstrated to confer poor prognosis on advanced NSCLC patients mainly due to an increase in metastatic burden [30]. These co-occurring lesions also engendered resistance against anticancer drugs in preclinical murine models of lung adenocarcinoma [31]. Studies in genetically engineered mice have shown that the simultaneous presence of *KRAS*^*G12D*^ mutation and deletion of *LKB1* in the lungs dramatically increases tumor burden and metastasis [31]. While many efforts have been made to understand the impact of individual genetic alterations, such as those in *KRAS* or *LKB1,* on cellular metabolism, very little is known about any influence on metabolism of the simultaneous presence of these two genetic alterations. The oncogenic cooperation between the KRAS^G12D^ mutant and loss of LKB1 expression was firstly characterized in pancreatic cancer, where it disturbed one carbon metabolism and incited epigenetic modifications thus supporting cancer growth [32]. In NSCLC, co-occurrence of mutant KRAS and LKB1 loss has been shown to impact on the urea cycle enzyme CPS1 providing an alternative pool of carbamoyl phosphate to maintain pyrimidine availability thus imposing a growth advantage on lung cancer cells [33]. Since both *KRAS* mutations and *LKB1* inactivating alterations affect cellular metabolism, it seems propitious to discern metabolic effects induced by the single oncogenic events from those elicited by their co-occurrence, with the ultimate aim to potentially exploit metabolic dependencies for novel therapeutic modalities. With these considerations in mind, we knocked-out the *LKB1* gene in well-characterized NSCLC cell clones harbouring KRAS wild type (WT) or mutant G12C proteins [16, 34]. We obtained an isogenic system in which *KRAS* mutation and *LKB1* inactivation were individually or concomitantly present. The effects of the genetic lesions individually or together on cell metabolism were investigated in these isogenic NSCLC cells by means of an integrated survey of proteomics, stable and dynamic metabolomics and functional in vitro strategies.

## Methods

### Cell culture, transfection and treatment

NCI-H1299-derived KRAS expressing clones have been obtained as previously described [35, 36]. In these clones, *LKB1* deletion has been achieved with CRISPR/Cas9 technology. Briefly, KRAS^WT^ and KRAS^G12C^ clones were transfected with all-in one CRISPR/Cas9 plasmid, containing both Cas9 cDNA and gRNA sequences, specific for *LKB1* gene (Sigma-Aldrich). After 24 h, cells were seeded at 1 cell/ml in Petri dishes, in order to obtain single-cell originated clones. Growing colonies were then detached using glass rings, expanded and checked for *LKB1* deletion by PCR and sequencing. Proteins from clones positive to *LKB1* editing were extracted and western blot analyses were performed with anti-LKB1 antibody, to verify LKB1 protein loss. The selected clones were tested also for the presence of modifications in the Cas9 off-targets (sequences in the genome with less than three mismatches compared to the Cas9 target sites) and they did not show any Cas9-induced variation in these sequences, thus confirming the lack of off-targets effects. Clones were maintained in RPMI 10% FBS added with 500 μg/ml of G418. Cells were routinely checked for mycoplasma presence by PCR.

For growth curve analyses, cells were seeded at 10000 cells/ml in triplicates and after 24 h they were counted every day with Multisizer counter (Beckman Coulter). Growth curves were plotted as the total number of cells at different time points and were the mean and standard deviation of two independent experiments. Doubling times were extrapolated from growth curves.

For 2-DG dose-response curve, cells were seeded in 96-well plates and, after 24 h, treated with increasing concentrations of 2-DG, dissolved in sterile-water and diluted in medium just before use. Seventy-two hours after treatment start, MTS assay was performed following manufacturer’s instructions. Dose-response curves were reported as percentage of cell viability compared to untreated controls and each data point consisted of at least six replicates. Three independent experiments were performed. The concentration inhibiting cell growth by 50% (IC50) values were extrapolated from the dose-response curves. IC50 values and 95% confidential intervals (CI) were calculated using Graphpad Prism, non linear regression (curve fit) analysis. Growth curve analyses in nutrient deprivation were performed by seeding the cells at the same concentration (30,000 cells/ml) in 96-well, blank plates in RPMI 1460 medium with 1% FBS, glutamine and glucose free, implemented with different glutamine and glucose concentrations.

Cell growth was analyzed with RealTime-Glo MT Cell Viability Assay (Promega). Briefly, at the time of the seeding, Real-time Glo reagents were added to the cells and luminescence was read with GloMax Instrument (Promega) 5, 22, 29 and 48 h after the time point “0”. Time point “0” was read 1 h after seeding, as suggested by manufacturer’s instructions. For each clone, Luminescence Units (LU) of the different time points were normalized on time ‘0’ LU. Growth curves were plotted as Normalized LU and represented the mean and SD of three independent replicates. Doubling times were calculated as described above.

### Label free-single reaction monitoring (SRM) based assay development

Single reaction monitoring (SRM) assay was developed following the overall strategy reported [37–39]. Forty seven metabolic enzymes involved in the major cellular metabolic processes spanning from glycolysis to fatty acid synthesis were chosen for their key role in the cellular metabolic route on the basis of both the scientific literature and the information stored in databases on protein function, such as UniProt (www.uniprot.org/) (Additional file 1: Table S1). Four to six unique peptides ranging from 6 to 20 amino acids in length containing tryptic ends, no missed cleavages, doubly or triply charged, were chosen for each of the selected proteins. All of the peptides containing amino acid Met, Trp, Asn and Gln were avoided and only selected when no other options were available [40]. Unique peptides observed in the whole cell proteomic analysis were prioritized during the peptide selection process. For those proteins for which no peptides were found in the whole proteomic analysis, prototypic peptides selection was based on “PeptideAtlas” (www.peptideatlas.org) information. All prototypic selected peptides were ranked by intensity using Skyline v 3.6 [41] and tested in SRM mode to select the most suitable transition for the quantification analysis. Two hundred (200) μg of protein extract (three biological replicates of each isogenic cell line) was submitted to digestion as reported above. Two (2) μg of peptides were analysed on a triple-quadruple mass spectrometer (Triple Quad 5500, AB SCIEX). Chromatographic separation was achieved with a 1200 HPLC Agilent technology equipped with a 10-cm Ascentist Express Peptide ES-C18 with a 2.1 mm inner diameter (Supelco). The peptide mixture was separated with a gradient from 2 to 60% acetonitrile in 24 min. In total, 293 peptides representative of 47 proteins were selected for quantification experiments. For 47 proteins, the optimal SRM transitions of the peptides with the highest signal-to–noise ratio in the fragment-ion were selected from the tested prototypic peptide panel (Additional file 2: Table S2 ).

### SRM peptide quantification by liquid chromatography-single reaction monitoring (LC-SRM)

SRM metabolic panels were measured in each isogenic cell clone (three biological replicates/clone) in time schedule SRM experiment. Four liquid chromatography-single reaction monitoring (LC-SRM) methods were used for the metabolic panel evaluation. At the end of the analysis transition groups corresponding to the targets peptides were extracted using Multiquant v 2.1 (SCIEX). SRM peaks were manually inspected by checking for co-elution, peak shape similarity, retention time. Only SRM peaks detected in two out of three biological experiments with a signal to noise ratio > 3 for the two top transitions were considered. Log2 peak area of each peptide was normalised against the mean of the area of the corresponding peptide among the different groups, in order to cope with technical variation. Abundance information of each protein among the experimental groups were obtained computing the sum of intensities of each peptide of a data protein [42, 43] (Additional file 3: Table S3). The sum of normalized peptide area values among isogenic cell line replicates showed a minimum median Pearson correlation coefficient of 0.80 (data not shown), demonstrating the experimental reproducibility among biological replicates. Statistical difference among experimental groups was evaluated using two-way ANOVA and Mann-Whitney-Wilcoxon Test (JMP pro 13, SAS).

### Metabolomics analysis: ^13^C labeling studies

NSCLC cell clones (1 × 10^6^ cells) were cultured in RPMI media supplemented with 10% dialyzed fetal bovine serum and 10 mM [U-^13^C]-labeled-glucose or 2 mM [U-^13^C]-labeled -glutamine (Cambridge Isotopes Laboratories). Cells were incubated 24 h for the ^13^C glucose and glutamine steady-state experiments and at different time points (1, 2, 4 and 8 h) for the ^13^C glucose and glutamine kinetics experiment. For the early ^13^C glucose incorporation, cells were incubated for 20 min. After labelling, cells were rinsed and metabolism quenched by liquid nitrogen. Metabolites were extracted using MeOH:ACN:Water (50:30:20) and incubated 20 min at − 80 °C. The lysates were centrifuged to remove precipitated protein, and 8 μl of supernatant were collected for liquid chromatography tandem-mass spectrometry (LC-MS/MS) analysis. Atlantis T3 column (3.5 μm, 150 × 2.1 mm, Waters) was used for LC separation and the detection of metabolites was performed using a Thermo Scientific LTQ-Orbitrap XL mass spectrometer with electrospray (ESI) ionization, examining metabolites in both positive and negative ion modes, over the mass range of 75–1000 m/z. The mobile phase for elution was a gradient established between water acidified with 0.1% formic acid (positive) 10 mM ammonium formiate (negative) (A) and acetonitrile (B) at a flow rate of 150 μl/min. Retention times of all metabolites of interest were validated using pure standards. The measured distribution of mass isotopomers was corrected for natural abundance of ^13^C. Relative metabolite abundance was calculated as percentage of total metabolite pool. For glucose and glutamine tricarboxylic acid (TCA) contribution, the sum of all isotopologues of the indicated metabolites was used. For ^13^C_6_-glucose labelling data interpretation M + 3 lactate, M + 3 alanine and M + 2 citrate derived from pyruvate were monitored. For ^13^C_5_-glutamine M + 4 citrate, succinate, fumarate and malate derived from glutamate were monitored. Representative isotopical trace labelling is shown in Additional file 4: Figure S1.

### Statistical analysis

Statistical analysis was done using both GraphPad PRISM v7 software and JMP Pro13. Briefly, when we compared two groups and one changing variable we used one-way ANOVA followed by Mann-Whitney-Wilcoxon test. For the experiments for which we analysed multiple groups and one changing variable, one-way ANOVA followed by Tukey-Kramer or Bonferroni was performed. When we compared two or more groups with more than one changing variable (for example, whole cell proteomics, SRM proteomics, untargeted metabolomics) we used two-way ANOVA.

## Results

### Growth properties of cells with different genetic lesions

**Fig. 1:**
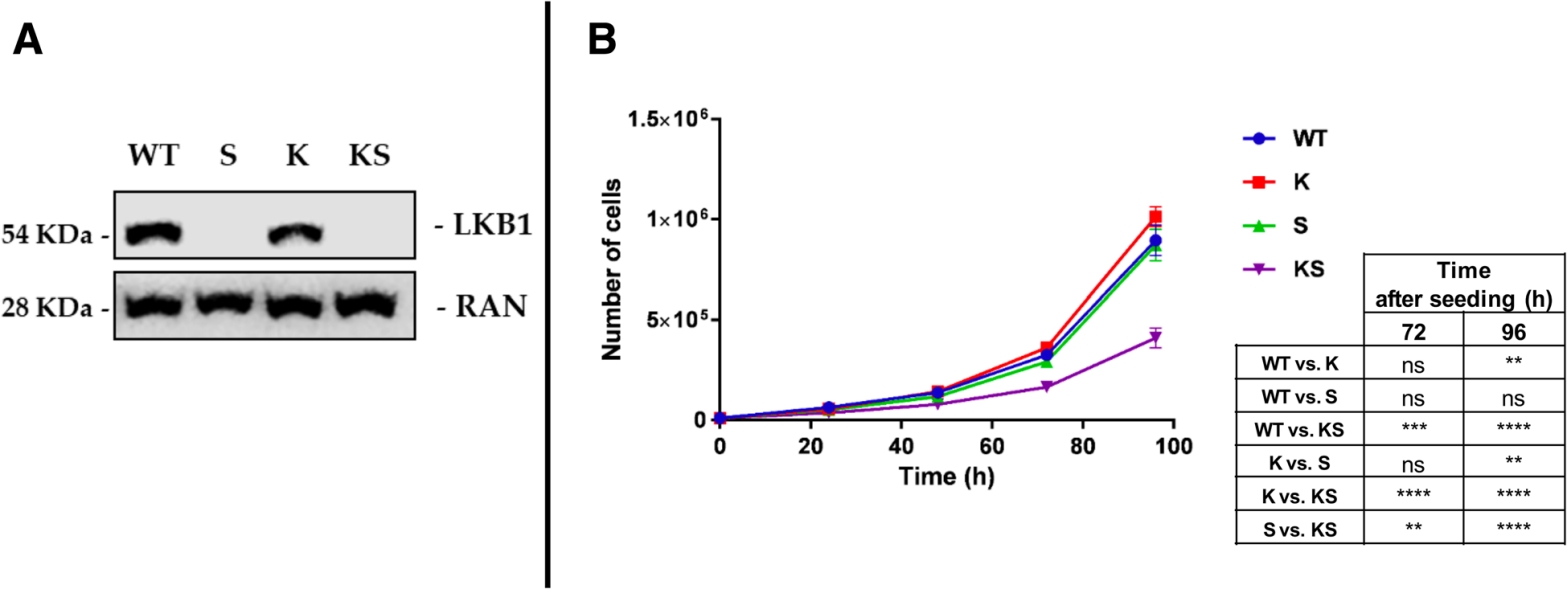
Molecular and cellular determinants. **a** Representative Western blot analysis performed at basal conditions, of LKB1 protein levels in all NSCLC NCI-H1299 isogenic clones (WT, S, K, KS). Ran was used as loading control. Three independent experiments were performed. **b** Growth curve of NSCLC isogenic clones. Cells were seeded at 10000 cells/mL in 6-well plates and counted every 24 h. Growth curves of the individual clones were plotted as number of cells at different time points and represented the average of three independent counts of three independent experiments. Statistical analysis was performed using two-way ANOVA test and Bonferroni post-test for multiple comparisons and is reported in the box below the figure. **p-value< 0.01, ***p-value< 0.001, ****p-value< 0.0001. No statistically significant differences were at 24 and 48 h

From our well-characterized NCI-H1299 derived clones expressing KRAS^WT^ or KRAS^G12C^ [35, 36], we generated two clones characterized by LKB1 loss taking advantage of the CRISPR/Cas9 technology, which allows locus-specific gene editing. As shown in Fig. 1a, the new clones resulted in LKB1 loss. In the following, the oncologic genotype combinations will be referred to as follows: KRAS^WT^/LKB1^WT =^ “WT”; KRAS^G12C^/LKB1^WT =^ “K”; KRAS^WT^/LKB1^loss^ = “S”; KRAS^G12C^/LKB1^loss^ = “KS”. Growth curve analyses performed on the clones revealed that KS impacted on in vitro cell growth. While the presence of a single genetic alteration did not alter cell growth, the cellular growth rate of the KS clone was reduced by 60% compared to WT (Fig. 1b). Similar results were obtained using another couple of independent S and KS clones. Based on this, we decided to perform further studies on single clones representative of the different genotype combinations.

### Whole cellular proteome of NSCLC clones harboring dual or single lesions shows differences in proteins mainly involved in metabolic modulation

**Fig. 2:**
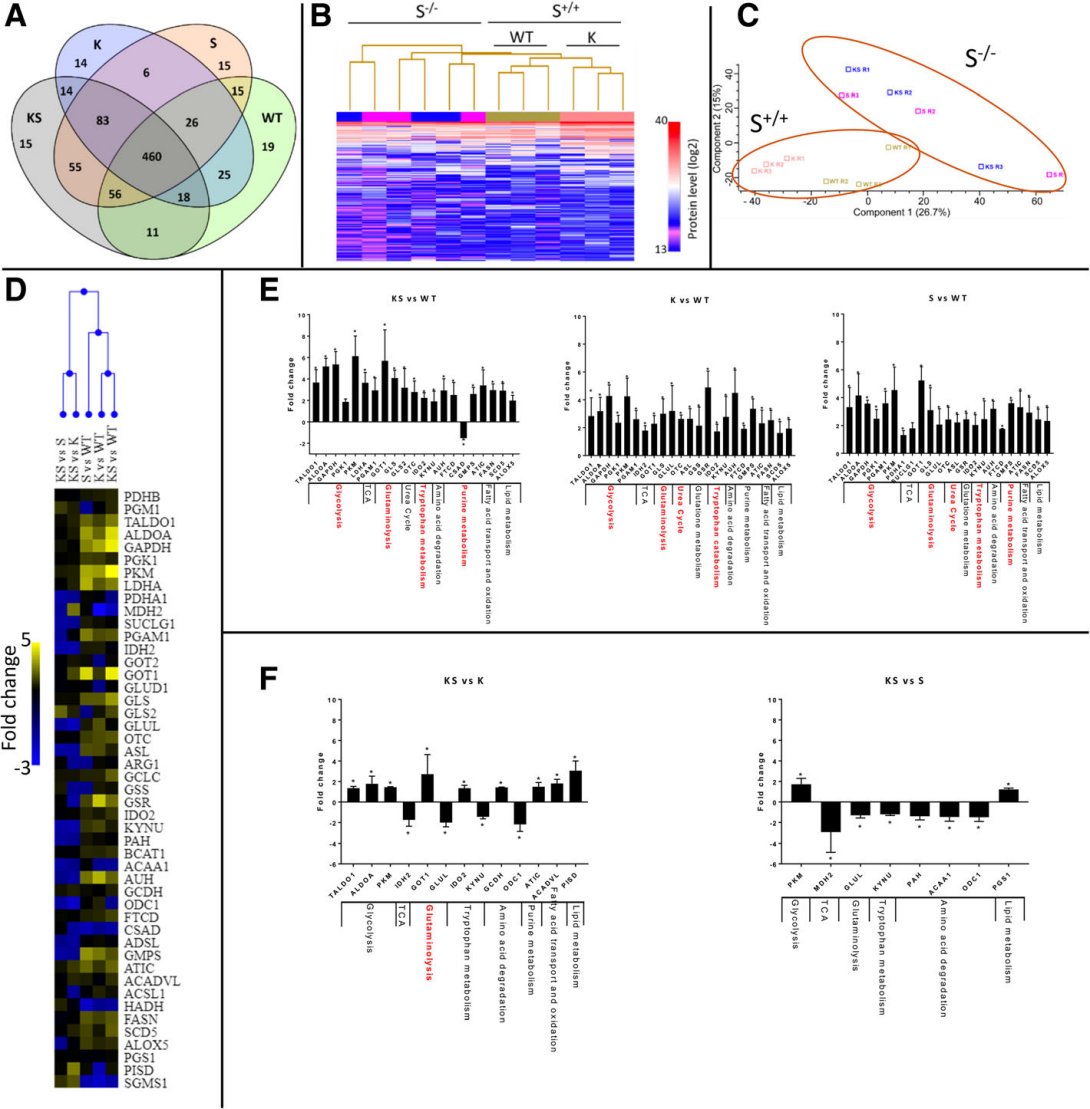
KS cells enclose/hold the metabolic enzymes induction triggered by the single lesions. **a** Venn diagram of the unique and shared identified proteins among the NSCLC NCI-H1299 isogenic clones harbouring WT, K, S and KS genetic determinants. **b** Heat-map display of unsupervised hierarchical clustering of the relative intensity (log2) of the identified proteins. **c** Principal component analysis (PCA) using the identified proteins. **d** Unsupervised hierarchical clustering of the fold change in abundance of metabolic enzymes identified by label Free-SRM targeted proteomics in NSCLC NCI-H1299 isogenic clones. **e** Fold change in abundance of the significantly altered metabolic enzymes detected through SRM label free analysis in NSCLC NCI-H1299 harbouring KS, K and S relative to WT clone. **f** Fold change in abundance of the significantly altered metabolic enzymes detected through SRM label free analysis in NSCLC NCI-H1299 harbouring KS relative to K or S clone. Columns represent protein fold change in abundance (mean ± SD, 3 biological replicates). Red highlighted the most affected metabolic pathways based on both the number of significantly deregulated proteins relative to the number of overall monitored proteins by SRM target proteomic strategy and on the fold change of abundance (± two-fold changes). Statistical analysis was performed using two-way ANOVA and Mann-Whitney-Wilcoxon Test *p-value< 0.05, ***p-value< 0.001

A single run label-free mass spectrometry (MS) proteomics workflow [44] of all isogenic cell clones identified 832 proteins. Among these, 460 proteins (55%) were common across all clones, only a few proteins (1.8%) were found to be unique to each isogenic cell type (Fig. 2a and Additional file 5: Table S4). Unsupervised hierarchical clustering of the 832 proteins resulted in two main groups distinguished by the S expression status. Inside the S expressing group, there was a sub-cluster based on K mutational status that was not present in the S group (Fig. 2b). Principal component analysis (PCA) supported the presence of two main protein groups the separation of which was based on S status, suggesting that the LKB1 expression status had a greater impact on the cellular proteome than the acquisition of KRAS mutation (Fig. 2c). In the cell clones with the differing genetic make-ups the following numbers of proteins were significantly altered relative to WT cells (Mann-Whitney-Wilcoxon test *p* < 0.05), KS: 180, K: 278 and S: 126, Only 31 proteins were commonly deregulated between KS, K and S cells (Additional file 4: Figure S2A). Functional annotation (PANTHER v10, www.pantherdb.org) of the deregulated proteins in the different clones suggests their involvement mainly in the following functions: KS - cellular component organization or biogenesis; K - cellular component organization or biogenesis, protein metabolism and folding, ferredoxin metabolism; S - RNA splicing (Additional file 4: Figure S2B, C, D and Additional file 6: Table S5). Considering the proteins commonly deregulated between the different data sets (Additional file 4: Figure S2A) significant overrepresentation was found for the 40 proteins shared between K and KS which were mainly involved in glycolysis. (Additional file 6: Table S5).

Biological annotation analysis converges to define a metabolic proteome modulation as the main signature characterizing both single and co-occurrent genetic lesions.

### Metabolic enzyme configuration in dual lesion as compared to the single lesion clones

In order to explore metabolic proteome alterations under the different genetic conditions we particularly monitored 47 metabolic enzymes involved in the major cellular processes from glycolysis to fatty acid synthesis (Additional file 1: Table S1). A label-free SRM-based proteomic approach was used. Unsupervised hierarchical clustering exploiting fold-changes of the selected enzymes revealed two clusters based on the molecular status of the reference counterparts (WT or single lesions K or S) (Fig. 2d). Univariate pairwise comparison (Mann-Whitney-Wilcoxon Test) revealed that the KS, K and S subtypes harbour altered enzymes belonging to glycolysis, glutaminolysis and tryptophan catabolism. The magnitude of the shifts in the KS, K and S groups were comparable, with ranges of fold changes of abundance of 2.2–6, 2.2–4.2 and 2–4.5, respectively. Only the S and KS constellations impacted on purine metabolism, while K and S displayed deregulated urea cycle components (Fig. 2e and Additional file 7: Table S6). KS harboured 13 deregulated enzymes relative to K clone. The abundances of 4 of these, GOT1, GLUL, ODC1 and PISD, differed two-fold or more from those in K. The first two enzymes are involved in the synthesis of glutamate and glutamine, fold-changes were 2.7 and − 2, respectively. ODC1 (fold-change − 2.2) is the first and rate-limiting catalyst of polyamine biosynthesis. PISD (fold-change 3) is engaged in phospholipid metabolism. In contrast, when compared to S, KS harboured 9 altered proteins, among which only MDH2, an enzyme involved in the TCA cycle, showed consistent deregulation with a fold change of − 2.9 (Fig. 2f and Additional file 7: Table S6). The comparison of KS with K and S suggests that co-occurrence of the two genetic lesions failed to generate major modifications in the metabolic enzyme asset either in terms of numbers or magnitude of enzymatic changes when compared to the cells with single lesions.

### Cellular metabolism in dual and single lesion clones

We wished to obtain a functional landscape of the activation state of the biochemical routes induced by KS. To that end, a broad-based metabolomic analysis was performed by combining untargeted (UT) and targeted (T) strategies (Additional file 8: Table S7). Functional enrichment analysis of the significant altered metabolites (pair-wise comparison, Mann–Whitney *p* < 0.05 relative to WT) suggests that KS had a greater impact on cell metabolism in terms of number of both deregulated pathways and mapped metabolites than K or S. Deregulation of metabolites belonging to the urea cycle, ammonia recycling, methionine and arginine metabolism were observed. Notably, KS retained the main metabolic routes which were altered by the single genetic lesions, although abundance scores for each pathway were different when compared to WT cells (Additional file 4: Figure S3A). The capability of KS to differentially modulate biochemical routes altered in cells harbouring the single genetic lesions was further highlighted by the differential abundance scores for the significantly enriched pathways (pair-wise comparison, Mann–Whitney *p* < 0.05 relative to K or S) (Additional file 4: Figure S3B).

**Fig. 3:**
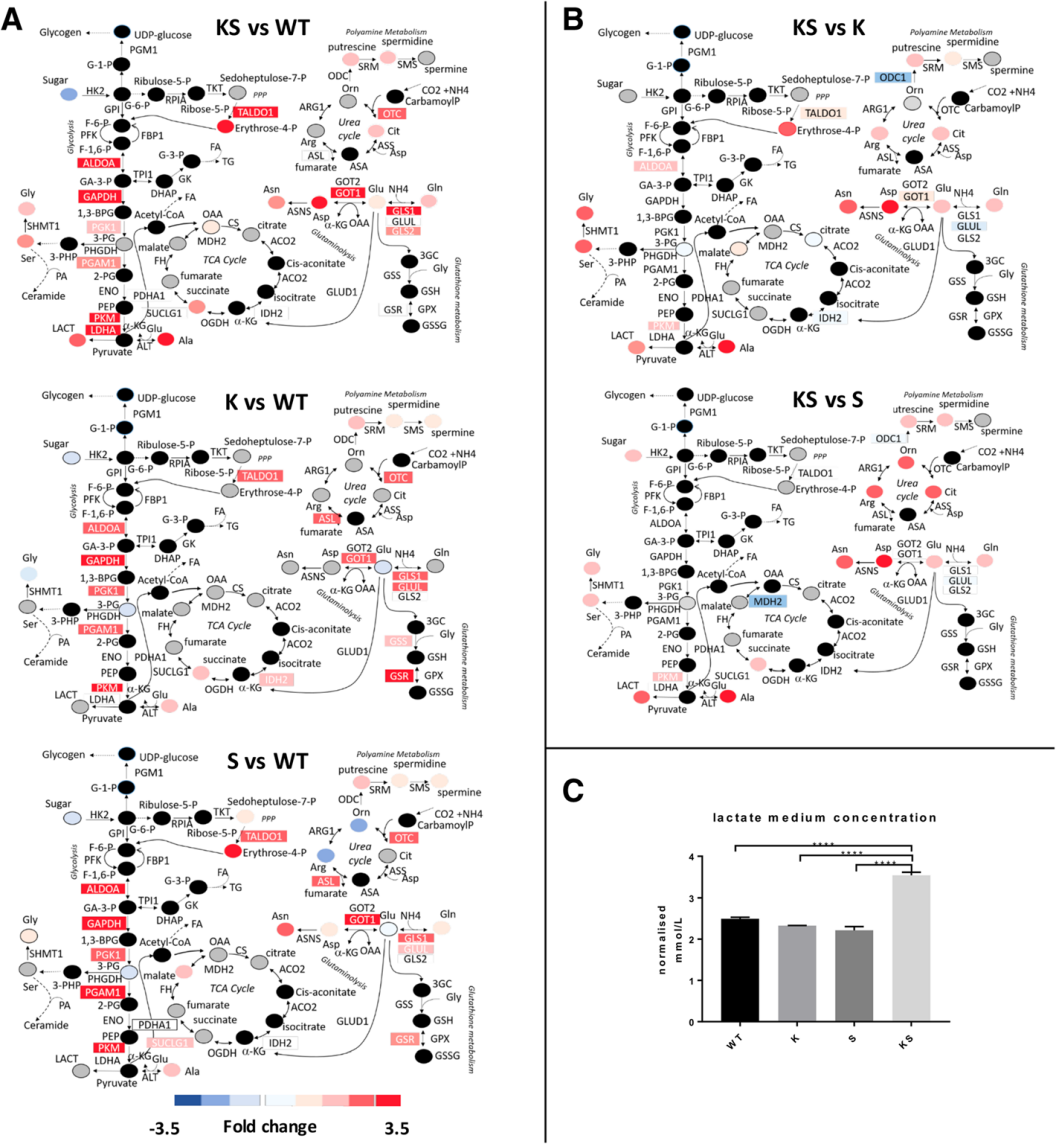
KS enhanced the exploitation of the central cellular metabolism compared to single lesions. **a** Central cellular metabolism alteration in NSCLC NCI-H1299 isogenic clones harbouring KS, K and S relative to WT. **b** Central cellular metabolism alteration in NSCLC NCI-H1299 harbouring co-occurring KS relative to single K or S lesions. Measured proteins and metabolites are labelled as color-coded rectangle and circle respectively. Colours correspond to the fold-change in abundance relative to the parental counterpart (WT): red, increase; blue, decrease; black, not measured; grey, measured but not statistically significant. Metabolites and enzymes were reported using standard abbreviation. **c** Extracellular lactate concentrations determined in cultured conditioned medium 48 h after cells seeding. Data are expressed as cell-normalized mmol/L ± SD, n: 3 biological replicates. Significant differences were computed by one-way ANOVA and Tukey’s multiple comparisons test (GraphPad Prism, V7.02) ***p-value< 0.001

By combining our multilayer data with known metabolic networks, we assembled a metabolic map (Fig. 3) depicting the distribution of changes in the abundance of proteins or metabolites for the most enriched/interconnected pathways altered by KS, K or S. Figure 3a shows comparable up regulation of the glycolytic enzymes ALDOA, GAPDH, PGK1, PGAM1, PKM and LDHA in all subtypes relative to WT. However, under KS conditions glycolytic end-products lactate (lact) and alanine (ala) were accumulated when compared to K or S (Fig. 3a, b). Increased intracellular lactate in KS cells was reflected by lactate accumulation in the medium (Fig. 3c), reflecting highly active glycolysis. GLS1 was upregulated in KS, K and S cells, whilst the glutaminolytic substrate glutamate (glu) was upregulated only in KS but not K or S cells (Fig. 3b). There were no major alterations related to the TCA cycle in KS relative to WT, K or S cells. (Fig. 3a, b).

All genetic alterations impacted on the non-oxidative phase of the pentose phosphate pathway (PPP) as evidenced by up-regulation of the TALDO1 protein. Changes in its by-product (erythrose 4-p) was observed only in KS and S cells. Serine (ser) and glycine (gly) were significantly raised under KS conditions but only modestly affected by the single lesions (Fig. 3a).

**Fig. 4:**
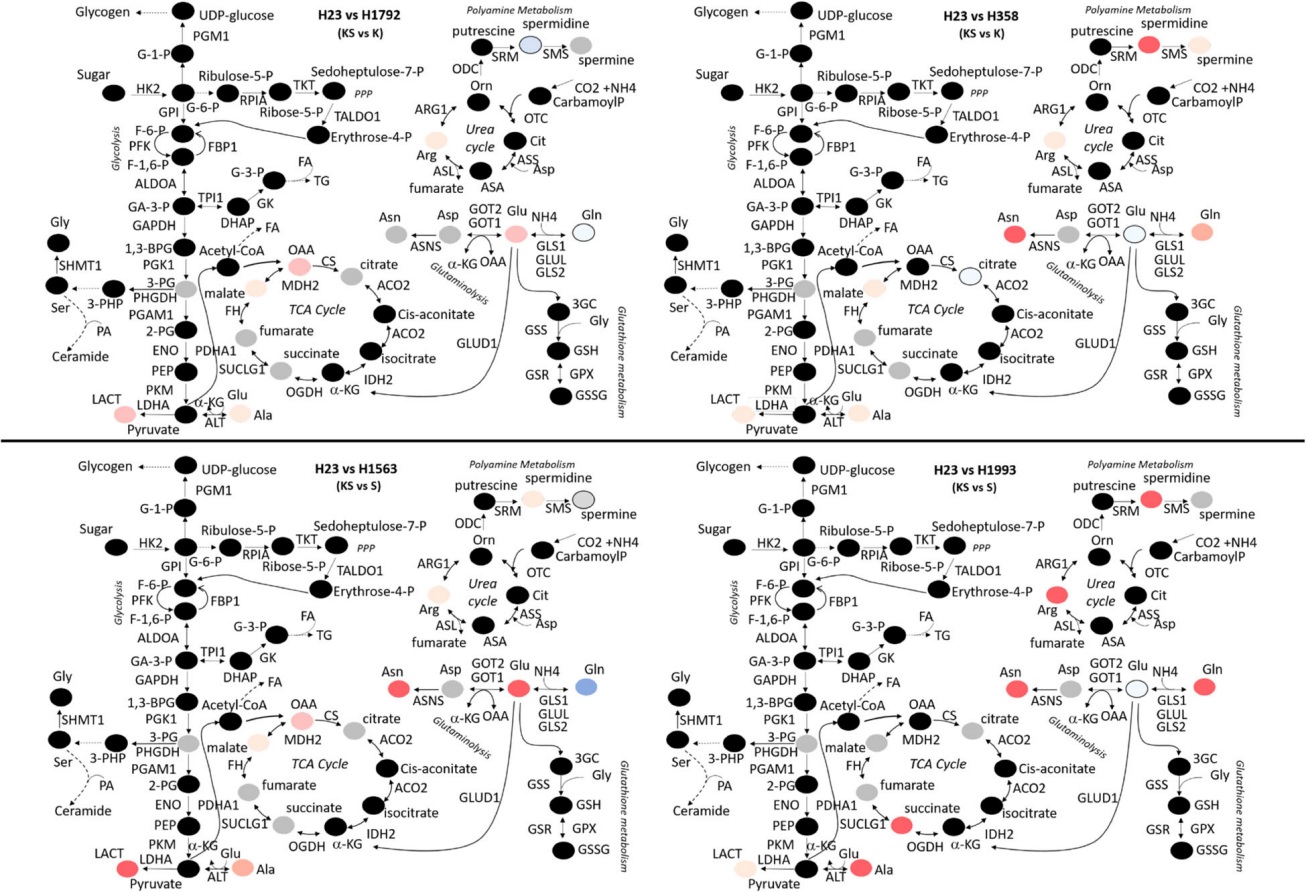
Central cellular metabolism alteration in lung cancer cell lines endogenously harbouring these oncogenic alterations: H23 (KS), H1792 (K), H358 (K) H1563 (S) and H1993 (S). Significant metabolites differences were computed using Mann-Whitney-Wilcoxon Test). Measured metabolites are labelled as color-coded circle. Colours correspond to the fold-change in abundance relative to the single K or S: red, increase; blue, decrease; black, not measured; grey, measured but not statistically significant. Metabolites are reported using standard abbreviation

The two urea cycle enzymes OTC and ASL were upregulated in all clones, although the former significantly only in KS. The induction of urea cycle enzymes was associated with a change in metabolites. Arginine (arg) and ornithine (orn) were increased in S, and citrulline (cit) in KS cells. Putrescine, spermidine and spermine, metabolites germane to polyamine metabolism, were increased under all three genetic conditions (Fig. 3a). Proteomic and metabolomic data integration highlighted that cells with both lesions, whilst being characterized by the same central cellular metabolic routes of their single lesion counterparts, were able to exploit such routes through a heightened metabolites production. To explore whether the metabolic landscape observed in our isogenic clones was also present in endogenously mutated lung adenocarcinoma cell lines, we analysed the metabolic profiling of five cell lines namely H23 (KS), H1792 (K), H358 (K) H1563 (S) and H1993 (S). We confirmed that alterations in the glycolytic end-product lactate and the glutaminolytic substrates glutamine (gln) and glutamate (glu) under KS (H23) rather than K (H1792, H358) or S (H1563, H1993) genetic configuration occurred also in these lung adenocarcinoma cell lines (Fig. 4).

### Differences in use of the glycolytic and glutaminolytic axes as carbon source between dual and single lesion clones

**Fig. 5:**
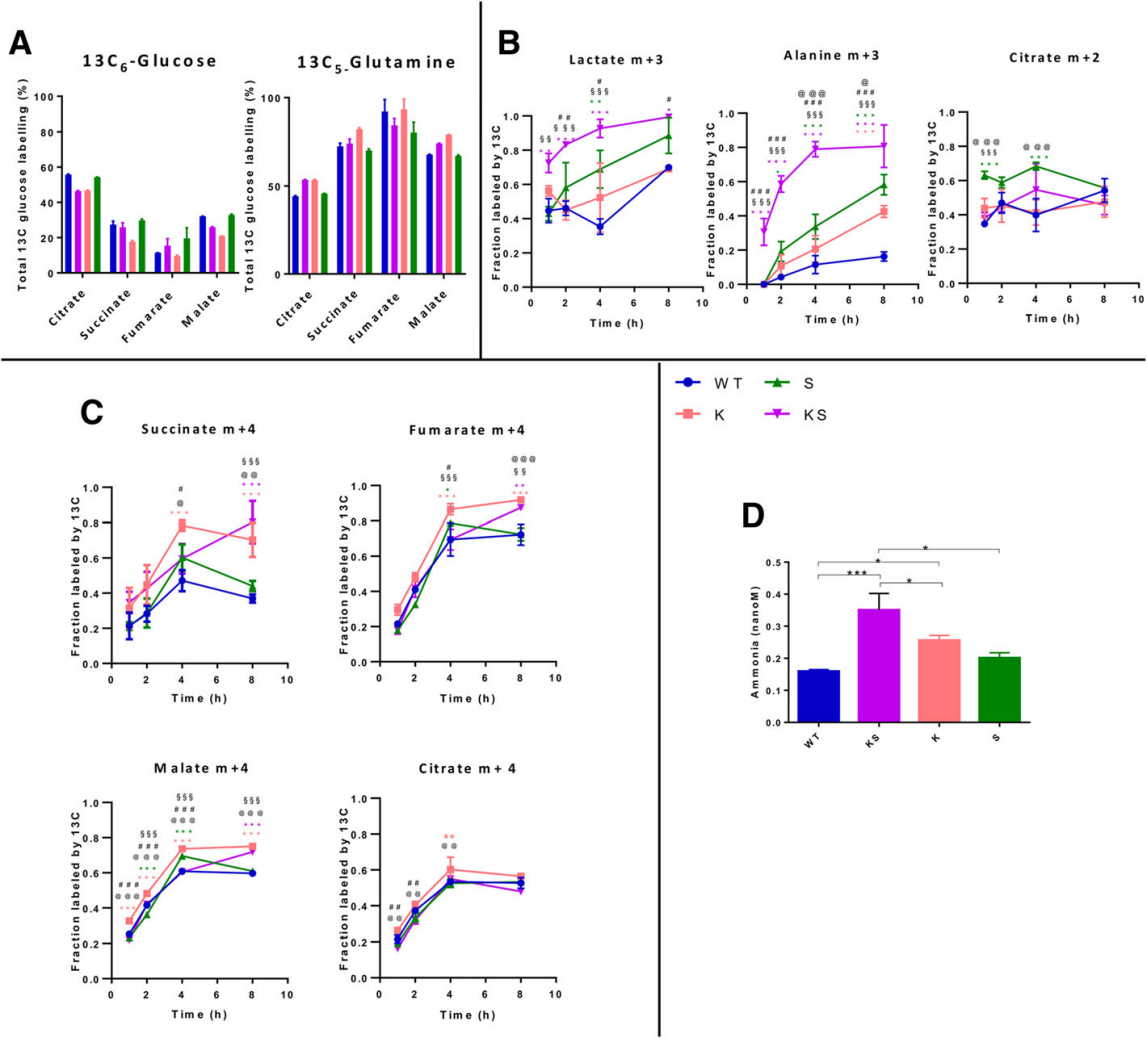
KS enhanced the glucose and glutamine metabolic flux. **a** Percentage of ^13^C incorporation into TCA cycle intermediates after addition of ^13^C-glucose and ^13^C-glutamine (steady state, 24 h of labelling) in NSCLC NCI-H1299 derived clones KS, K, S and WT, respectively. **b** Kinetic analysis of glucose incorporation in presence of KS, K, S and WT genetic backgrounds in NSCLC NCI-H1299 incubated with ^13^C-glucose after 1, 2, 4, 8 h. **c** Kinetic analysis of glutamine incorporation in presence of KS, K, S and WT backgrounds in NSCLC NCI-H1299 incubated with ^13^C-glutamine after 1, 2, 4, 8 h. M + 2, + 3 or + 4 labeled compounds indicate molecules of those compounds that contain 2, 3 or 4 13C atoms, respectively. **d** Ammonia release (nM) in conditioned medium in presence of KS, K, S and WT backgrounds in NSCLC NCI-H1299 after 48 h from cell seeding. *p*-values were calculated using one-way ANOVA test and Tukey Kramer post-test for multiple comparisons (GraphPad Prism, V7.02). Significant differences are marked as * vs WT; # KSvsK; § KSvsS; @ KvsS The number of symbols refer to the level of significance: one *p* < 0.05; two *p* < 0.01, three *p* < 0.001

To further decipher changes in metabolic state triggered by the single and the dual genetic lesions, we performed metabolic tracing studies with ^13^C isotope labeled glucose [U-^13^C_6_] and glutamine [U-^13^C_5_]. In all clones ^13^C-derived glucose or glutamine were readily converted to pyruvate or glutamate which were then incorporated into TCA cycle intermediates, indicating active oxidative phosphorylation independent of genetic backgrounds (Fig. 5a). Specifically, about 50% of the ^13^C-derived glucose carbon was recovered from citrate, an early step component of the TCA cycle, whilst about ~ 25% were incorporated into later step components, such as succinate, fumarate and malate. Indeed, 75% of the ^13^C-derived glutamine carbon enriched latter TCA intermediates, and contributed to the remaining ~ 50% recovered from citrate, indicating that glutamine and not glucose, supports in the main the latter steps of the TCA cycle and maintains mitochondrial oxidative phosphorylation (OXPHOS) on glucose-independent feeding.

Kinetic profiling analysis of incorporation of ^13^C-derived glucose or glutamine after incubation for 1 to 8 h showed a prominent use of both glucose and glutamine as source of carbon under KS conditions when compared to K, S or WT. Notably, production of both lactate and alanine was increased in the KS as compared to K or S clones (two-way ANOVA, Tukey’s multiple comparisons test) suggestive of differential extent of glycolysis (Fig. 5b). To investigate in depth the metabolism of glucose in KS clones we analysed the isotopic enrichment of ^13^C-glucose after 20 min. Intracellular ^13^C-glucose and labelled lactate and alanine derived from it were significantly increased in KS and S relative to K and WT clones (Additional file 4: Figure S4). Therefore KS clone retained the property of S cells to incorporate glucose and exploit the glycolytic axis.

In all clones ^13^C glutamine-derived TCA metabolites corroborated functional TCA cycling to maintain oxidative phosphorylation and anabolic processes. The kinetic profile of ^13^C glutamine indicates that the K and KS clones exploited the latter part of the TCA cycle more robustly than the S or WT clones, as reflected by a higher level of labelled succinate, fumarate and malate in the former (Fig. 5c). Citrate derived from labelled glucose or glutamine was altered in S and K but not KS clones (Fig. 5b, c). Ammonia production was increased in KS and K relative to S or WT cells, indicative of increased glutaminolysis (Fig. 5d). Mitochondrial and glycolytic functionalities under stress were determined using the XF Glycolysis and Mito Stress Test in all the isogenic clones (WT, K, S, KS) assayed under the same oxygen culturing conditions. KS cells were characterised by lower spare respiratory capacity after exposure to mitochondrial stressors compared to K or S cells (Additional file 4: Figure S5A, B). Moreover, lower glycolytic capacity and reserve was featuring KS compared to K clones (Additional file 4: Figure S5C, D). These findings suggest differential abilities of KS cells in responding to ATP demands under conditions of mitochondrial and glycolytic stress.

### Effect of glycolysis inhibition and nutrient deprivation on cell growth

**Fig. 6:**
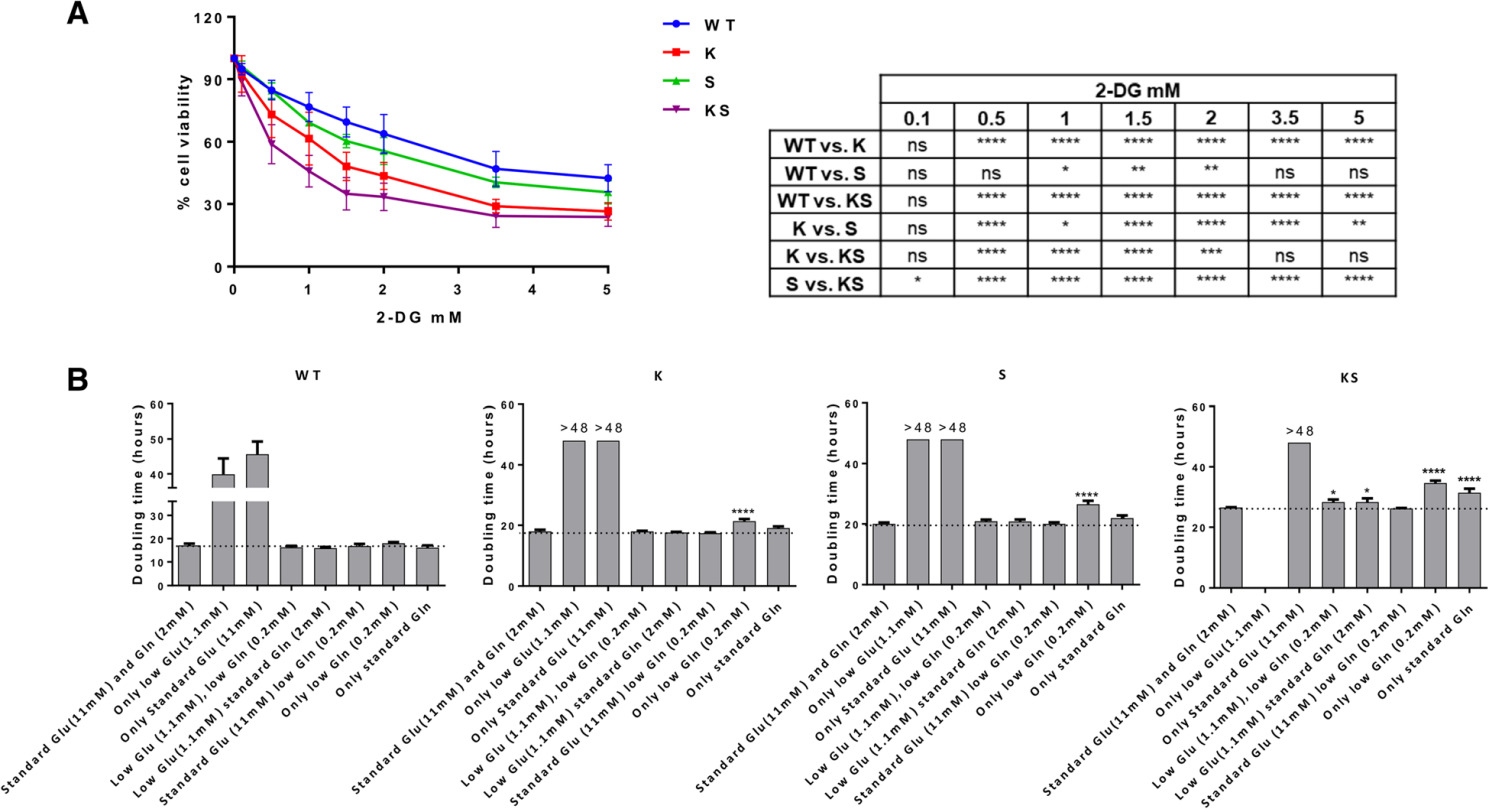
Functional evaluations converge to define the major susceptibility of KS to metabolic impairment. **a** Response of NSCLC NCI-H1299 derived clones treated with increasing concentrations of 2-DG for 72 h, detected by MTS assay and plotted as dose-response curves. The mean of four independent experiments and SD are shown. Statistical analysis was performed using two-way ANOVA test and Bonferroni post-test for multiple comparisons and is reported in the box. **b** Doubling times (hours) of KS, K, S and WT NSCLC NCI-H1299 clones cultured in different nutrient combinations. Doubling times were extrapolated from growth curves (Additional file 4: Figure S6) and represented as mean and SD of three independent replicates. Doubling times with a positive value higher than 48 h (the last measured time point) were expressed as > 48. Negative values were not reported. Dashed line highlights standard growth rate. Statistical analysis was performed using one-way ANOVA test and Bonferroni post-test for multiple comparisons. For each clone, each doubling time obtained in the different nutrient-deprived conditions was compared to the doubling time of standard culture conditions. * *p*-value < 0.05, **** *p*-value < 0.0001

Given the differences in exploitation of the energetic axis between KS and S or K clones, we investigated their metabolic vulnerability to metabolic stress. The viability of KS cells was more affected by perturbation of the glycolytic pathway via treatment with 2-deoxy glucose (2-DG) than that of K, S or WT cells (Fig. 6a). Respective IC50 values (in mM) were 0.8412 (C.I. 0.7583–0.9331), 1.496 (C.I. 1.366–1.639) 2.472 (C.I. 2.336–2.617) and 3.461 (C.I. 3.143–3.811).

To corroborate the differences in dependencies on energy sources between the cells, growth was studied using media containing different glucose (glu)/glutamine (gln) combinations: either standard, absent (0%) or low (10%) (Fig. 6b and Additional file 4: Figure S6). Without glucose and glutamine neither clone was viable. Glutamine-free conditions were detrimental to all clones even in the presence of glucose, although WT cells were able to replicate within 48 h under these conditions. These results show that glutamine was essential for cell growth and survival. Under conditions of low (1.1 mM) glucose, low (0.2 mM) or standard (2 mM) glutamine were sufficient to rescue K and S cells growth at their standard rate, whilst this rescue does not seem to occur, or to occur at lower extent in KS clones. In incubates with standard (11 mM) glucose and low glutamine, KS cells grew at their standard rate. Absence of glucose in the presence of low (0.2 mM) or standard (2 mM) glutamine reduced growth in K and S clones when compared to their standard rates. This phenomenon was more evident in KS clones. Overall KS clone displayed a statistically significant growth defect in presence of energetic stress caused by both glycolysis inhibition and by nutrients limitation (glucose/glutamine) than those harboring single oncogenic lesions and the parental clones.

## Discussion

Here we investigated how concomitant *KRAS* mutation and *LKB1* loss in NSCLC-NCI-H1299 clones affect cellular metabolism by systems-level analysis combining metabolic enzyme abundance survey with static and dynamic metabolic profiling data. Cells with either single lesion showed active energy metabolism via exploitation of a functional glycolysis axis and an active mitochondrial machinery capable of oxidizing pyruvate and glutamine, although cells lacking LKB1 showed a preference for glycolysis whilst KRAS mutated cells preferred mitochondrial respiration, as previously reported [14, 22, 24]. We show that cells with both lesions, whilst being characterized by the metabolic traits of their single lesion counterparts, were able to exploit these metabolic routes through a heightened metabolites production. This metabolic ability was characterizing not only our KS isogenic clone but also the endogenously mutated adenocarcinoma cell lines H23. Enhanced metabolic production was particularly prominent with regards to the glycolysis end-products lactate and alanine and the glutaminolyic substrate glutamate despite the same magnitude of induction in metabolic enzymes as in the single lesion. The mismatch between the enzyme level and the metabolite abundance is in line with the reported role of protein post-translational modifications (PTMs) in regulating enzyme activity and the response to changes to external conditions or internal states [45, 46].

Using detailed analyses of ^13^C isotopologues of citrate, succinate, fumarate, and malate, we uncovered for the first time in NSCLC cells a glucose-independent glutamine-driven TCA cycle under conditions of standard glucose concentration in the medium. Cells directed glucose-derived carbon skeletons mainly towards citrate synthesis, while glutamine-derived succinate, fumarate and malate drove the latter steps of the TCA cycle for ATP production and supported citrate production to fuel anabolic processes. Until now a glucose-independent TCA cycle in the presence of normal glucose has been identified only in haematological cancer cells [47, 48]. Glucose-independent TCA feeding was an intrinsic characteristic of all genetic backgrounds investigated here (i.e. KS, K, S, WT), whilst entry of glutamine, after conversion to glutamate, into the TCA cycle and its oxidation to succinate, fumarate and malate were facilitated in cells with *KRAS* mutation (K) or both lesions (KS). These data, together with the observed increase in ammonia production, suggest more efficient glutaminolysis in KS and K than in S or WT cells. Our observations are consistent with the findings of Kim et al. [33] which demonstrated how NSCLC cells with KS and K lesions rely on CPS1 protein addiction for proliferation. CPS1 controls the first step of the urea cycle, a reaction in which excess nitrogen containing compounds are incorporated into the cycle to be processed. The CPS1 addiction of KS cells might reflect an adaptive downstream metabolic configuration necessary to metabolize the ammonia pool generated from enhanced glutamine catabolism in the mitochondria.

**Fig. 7:**
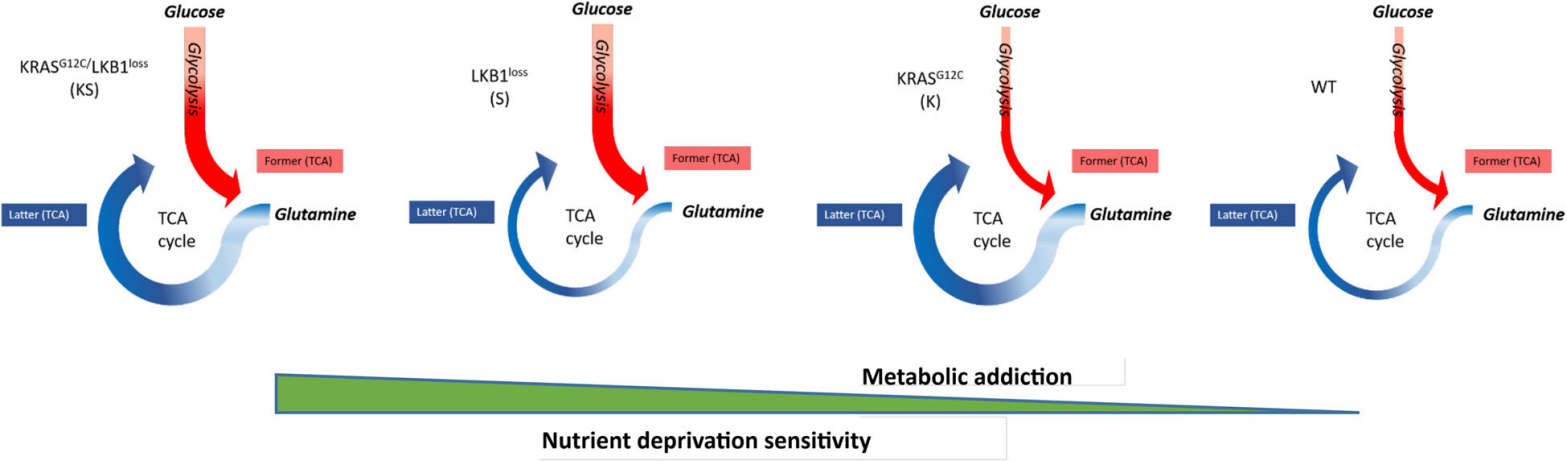
Schematic overview summing up our findings. Schematic diagram depicting the enhanced metabolic activity of KS co-occurrence in relation to its heightened susceptibility to nutrient limited conditions relative to single K or S and parental background in NSCLC H1299 derived clones. Red arrow thickness refers to the rate of glycolysis exploitation, blue arrow thickness refers to the rate of glutaminolytic cascade. Green triangle highlights the association between increased metabolic addiction with sensitivity to nutrient deprivation in NSCLC H1299 clones harbouring WT, S, K, and KS oncological genotypes

Most importantly, the greater rate of functionality of TCA and glycolysis in the KS cells was associated with both lower mitochondrial spare respiratory capacity (SRC) and lower glycolytic reserve. Since the SRC depicts the extra mitochondrial capacity of a cell to produce energy under stress conditions or increased cellular work [49–51] our observations, indicate that in the presence of double lesions (KS), cells achieve the maximum metabolic capacity, which cannot be further raised to compensate the increase ATP demand under OXPHOS and glycolytic restriction. The decreased metabolic plasticity in presence of co-occurring lesions was further demonstrated by the greater susceptibility towards survival impedance caused by 2-DG treatment or glucose/glutamine limitations. The increased metabolic rate demonstrated by us in the cells harbouring both genetic lesions relative to single lesion, failed to improve their cellular fitness, but instead was accompanied by reduced growth rates compared to those of WT or single lesion cells (graphically represented in Fig. 7), supporting the role of metabolic flexibility for long-term cellular survival and function [52]. Undoubtedly cells in culture lack the capabilities and properties imparted on them by the genuine in vivo environment, which can importantly modulate the metabolic phenotype of cancer cells [15, 53]. We previously demonstrated that metabolic traits observed in KRAS-mutated NSCLC cells in culture translate into a murine model of human NSCLC cell xenografts [54].

Patients with NSCLCs characterized by mutated KRAS in conjunction with LKB1 loss are associated with a progressively poor prognosis [55]. Nutrient starvation is an emerging strategy to reduce metabolite availability to tumor cells generating environments that can reduce the capability of cancer cells to adapt and survive and thus improving the effects of cancer therapies. In a wide range of animal cancer models multiple cycles of fasting cycles plus chemotherapy drugs promote differential stress sensitization, potentiate the activity of chemotherapeutics resulting in long-term cancer-free survival [56–58]. Our results hint at the possibility that energy stress induced by nutrient limitation via calorie restriction regimens or diets [59] may sensitize NSCLC tumors harbouring these lesions towards chemotherapy, thus potentially improving prognosis.

## Conclusions

Co-occurrence of *KRAS* mutation and *LKB1* loss in NSCLC cells induced an enhanced metabolic activity mirrored by a growth rate vulnerability under limited nutrient conditions relative to cells with the single oncogenetic lesions. This observation hints tentatively at the possibility that the findings presented here are indeed relevant also under conditions in vivo.

## Additional files

Additional file 1: **Table S1.** Metabolic enzymes and related pathways analysed by SRM. (XLSX 12 kb)

Additional file 2: **Table S2.** SRM peptides transitions. (XLSX 26 kb)

Additional file 3: **Table S3.** SRM peptides abundance. (XLSX 19 kb)

Additional file 4: Supplemental Methods and supplemental **Figure S1-S6**. (DOCX 1394 kb)

Additional file 5: **Table S4.** Protein list identified by label-free proteomics. (XLSX 176 kb)

Additional file 6: **Table S5.** Protein functional annotation. (XLSX 20 kb)

Additional file 7: **Table S6.** Fold-changes in abundance of metabolic enzymes. (XLSX 21 kb)

Additional file 8: **Table S7.** Statistically significant metabolites identified by untargeted and targeted metabolomics. (XLSX 52 kb)

## Abbreviations

2-DG: 2-deoxy glucose
AMPK: AMP-activated protein kinase
LC-MS/MS: Liquid chromatography tandem-mass spectrometry
LC-SRM: Liquid chromatography-single reaction monitoring
LKB1/STK11: Serine/threonine kinase 11 gene
MS: Mass spectrometry
NSCLC: Non-small-cell lung cancer
OXPHOS: Oxidative phosphorylation
PCA: Principal component analysis
PPP: Pentose phosphate pathway
PTMs: Protein post-translational modifications
SRC: Spare respiratory capacity
SRM: Single reaction monitoring
T: Targeted strategy
TCA: Tricarboxylic acid
UT: Untargeted strategy
WT: Wild type
