# Supplementary material for: Co-occurring KRAS mutation/LKB1 loss in non-small cell lung cancer cells results in enhanced metabolic activity susceptible to caloric restriction: an in vitro integrated multilevel approach
Source: J Exp Clin Cancer Res. 2018 Dec 4;37:302. doi: 10.1186/s13046-018-0954-5 (PMC6280460; doi:10.1186/s13046-018-0954-5)
Supplement: Supplementary file 4 — Supplemental Methods and supplemental Figure S1-S6. (DOCX 1394 kb) [file 13046_2018_954_MOESM4_ESM.docx]

**Co-occurring** **KRAS mutation/LKB1 loss in non-small cell lung cancer cells results in enhanced metabolic activity** **susceptible to caloric restriction: an in vitro integrated multilevel approach**

Elisa Caiola^2^, Francesca Falcetta^3^, Silvia Giordano^1^, Mirko Marabese^2^, Marina C. Garassino^4^, Massimo Broggini^2^$, Roberta Pastorelli^1^$, Laura Brunelli^1^$#

**Supplementary Methods**

**Whole cell proteomics sample preparation and Data analysis**

Total cell proteins were extracted from four isogenic cell clones (three biological replicates/clone) using RIPA buffer (150 mM NaCl, 1.0% Triton, 0.5% sodium deoxycholate, 0.1% SDS, 50 mM Tris, pH 8.0) and equal amount of proteins (30 μg) for each sample was submitted to in-solution digestion. Briefly, proteins were reduced with 10 mM dithiotreitol (DTT) at 56 °C for 1h and then alkylated with 55 mM iodoacetamide (IAA) for 50 min at room temperature. Proteins were then digested overnight with trypsin at a concentration of 1:50 (w/w). After digestion, peptides were loaded on SampliQ C18 ODS cartridge (Agilent technologies) and eluted following manufacturer’s instruction. Peptides were separated on a Thermo Scientific Biobasic 18 column (150 × 0.18 mm ID, particle size 5 µm) coupled online to a LTQ Orbitrap XL (Thermo Scientific) via a DESI Omni Spray (Prosolia) used in nanospray mode. Peptides were eluted with a 240 min gradient of 5%–60% buffer B (80% ACN) at a flow rate of 2 μL/min. The LTQ Orbitrap XL was operated in a data dependent mode with a survey scan range of 400-2000 m/z and a resolution of 60’00 in parallel with low-resolution MS/MS scans of the ten most abundant precursor ions with a charge ≥ 2. Dynamic exclusion of sequenced peptides was set to 30 s to reduce the number of repeated sequences. Data were acquired using the Xcalibur software (Thermo Scientific). MaxQuant software (version 1.5.3.30) was used to analyze MS raw files [1]. MS/MS spectra were searched against the human Uniprot FASTA database (Version 2016) and a common contaminants database (247 entries) by the Andromeda search engine [2]. Cysteine carbamidomethylation was applied as fixed and methionine oxidation as variable modification. Enzyme specificity was set to trypsin with a maximum of two missed cleavages and a minimum peptide length of 7 amino acids. A false discovery rate (FDR) of 1% was required for peptides and proteins. Peptide identification was performed with an allowed initial precursor mass deviation of up to 7 ppm and an allowed fragment mass deviation of 20 ppm. Protein identification required at least 1 razor peptide. A minimum ratio count of 1 was required for valid quantification events via MaxQuant’s Label Free Quantification algorithm (MaxLFQ) [1]. In total, our analysis resulted in 5470 unique peptides corresponding to 1349 distinct proteins at a peptide and protein false-discovery rate (FDR) of less than 1%. Data were filtered for common contaminants and peptides only identified by side modification were excluded from further analysis. In addition, it was required to have a minimum of two valid quantifications values in at least one group of replicates. Bioinformatic analysis was performed in the Perseus software environment [3]. Statistical analysis of protein changes was performed on log2 transformed intensities for those values that were found to be quantified in out of two biological replicates/clone applying two-way ANOVA and Mann-Whitney-Wilcoxon Test selecting cut off p<0.05 (JMP v 13).

The whole mass spectrometry proteomics data have been deposited to the ProteomeXchange Consortium (http://proteomecentral.proteomexchange.org/cgi/GetDataset) via the PRIDE partner repository with the data set identifier PXD007838. All other data supporting the findings of this publication are available within the article and its supplemental information files.

Comprehensive biological process analysis of the significant proteins on each data set were conducted using the PANTHER Classification System and the statistical overrepresentation tool (http://www.pantherdb.org/) [4]. The binomial test was applied to determine whether there was a statistical overrepresentation of the proteins in the input list relative to the reference Homo Sapiens genome, on the basis of PANTHER classification categories. Bonferroni-corrected p-values < 0.05 were considered significant.

**Metabolomics analyisis**

**Untargeted metabolomics approach (FIA-QTOF-MS/MS)**

Metabolites were extracted as previously reported in Brunelli et al [5] using equal number of cells for each isogenic clone. The analysis was performed on an Agilent 1290 infinity Series coupled to an Agilent 6550 iFunnel Q-TOF mass spectrometer (Agilent) equipped with an electrospray source operated in negative and positive mode. The flow rate was 150 μL/min of mobile phase consisting of isopropanol/water (60:40, v/v) buffered with 5 mM ammonium at pH 9 for negative mode and methanol/water (60:40, v/v) with 0.1% formic acid at pH 3 for positive mode. Reference masses for internal calibration were used in continuous infusion during the analysis (m/z 121.050873, 922.009798 for positive and m/z 11.9856, 1033.9881 for negative ionization). Mass spectra were recorded from m/z 50 to 1100. Source temperature was set to 320°C with 15 L/min drying gas and a nebulizer pressure of 35 psig. Fragmentor, skimmer, and octopole voltages were set to 175, 65, and 750 V, respectively. MS/MS fragmentation pattern of the significantly features were collected and used to confirmed metabolite identity. Before each sample a blank sample (isopropanol/water (60:40, v/v) negative, methanol/water (60:40, v/v) with 0.1% formic acid positive) was run to minimize the carry-over effect. This method allows a rapid metabolic profiling of polar and non polar compounds with the exclusion of lipid classes, which were not considered in untargeted data elaboration due to the intrinsic method limitation in the discrimination of isobaric forms.

All steps of data processing and analysis were performed with Matlab R2016a (The Mathworks, Natick) using in-house developed script following the workflow proposed by Fuhrer [6]. Centroid m/z lists were exported to csv format. Briefly, in this procedure, we first subtracted from each sample its relative blank sample to minimize the carry-over effect, then we applied a cut-off to filter peaks of less than 500 ion counts for negative and 1000 ion counts for positive ionization to avoid detection of features that are too low to be statistically significant. Centroid m/z lists from different samples were merged to a single matrix by binning the accurate centroid masses within the tolerance given by the instrument resolution (about 10 ppm). The output m x n matrix contains the m peak intensities of each mass for the n analyzed samples. Because mass axis calibration is applied online during acquisition, no m/z correction was applied during processing to correct for potential drifts.

Output m/z list was submitted to statistical analysis (two-way ANOVA and Mann-Whitney-Wilcoxon Test (JMP pro 13, SAS).) in order to select features with a statistical significance between groups of comparison. Significant altered features were identified by database searches (HMBD, METLIN, http://www.hmdb.ca/, http://metlin.scripps.edu) in positive and negative ionization considering only protonate/deprotonate ion. Definite identifications were reported only for metabolites with accurate mass match <10 ppm and a MS/MS fragmentation patterns similarity >99% relative to reference compound present on the database.

**Targeted metabolomics analysis**

A targeted quantitative approach using AbsoluteIDQ 180 kit, Biocrates was applied as previously published [7] for a targeted analysis on a aliquot (30 ul) of cell metabolite extracts used for the untargeted analysis. Significant metabolite changes were evaluated using two-way ANOVA and Mann-Whitney-Wilcoxon Test (JMP pro 13, SAS).

**Metabolic pathway analysis**

For biological interpretation of the metabolite dataset, we mapped the significant metabolites having a unique HMBD identifier to the KEGG pathway database (Kyoto Encyclopedia of Genes and Genomes; (www. genome.jp/kegg/), using MetaboAnalyst 3.0, a comprehensive online tool suite for metabolomic data analysis and interpretation (www.metaboanalyst.ca).

Since phosphatidylcholines (PC), lysophosphatidylcholines (LPC) and sphingomyelins (SM) have isobaric and isomeric forms that give several HMDB identifiers, lipids were excluded from enriched analysis. Enrichment analysis (EA) tools were used to identify metabolic pathways that were most likely to be associated with the different oncogenetic lesions. For data interpretation we considered only those metabolic pathways with at least three mapped metabolites. Differential abundance score was calculated for each significant enriched pathway as reported by Hakimi [8]. Such score captures the tendency for a given metabolite in a pathway to be increased/decreased relative to the counterpart condition. A score of 1 indicates all measured metabolites in the pathway are increased and -1 all measured metabolites in the pathway are decreased. When an identified metabolite was found significantly altered in both untargeted and targeted metabolomics analyses, then the targeted quantification value was considered for the subsequent data interpretation.

**Oxygen consumption rate (OCR) and extracellular acidification rate (ECAR) measurements**

OCR and ECAR measurements were monitored using a Seahorse XFp extracellular flux analyser (Agilent) to evaluate mitochondrial and glycolytic function through mitochondria stress test and glycolysis stress test following manufacturer’s instructions. Cells were plated at 20000 cells/well in a 8-well Seahorse plate with two wells per row of the culture plate containing only supplemented media without cells, as a negative control. Oligomycin (1 μM), Carbonyl cyanide-4-(trifluoromethoxy) phenylhydrazone (FCCP, 1μM) and Rotenone/antimycin A (0.5 μM) were used for the mitochondria stress test. Glucose (10 mM), Oligomycin (1 μM) and 2-DG(50 mM) were used for the glycolysis stress test. Data are expressed as median ± SEM, n: six biological replicates.

**Lactate level determination in cell medium**

Extracellular lactate concentrations (mmol/L) were measured in cultured conditioned media using Vi-Cell MetaFlex (Beckman Coulter) 48h after cells seeding (three biological replicates/clone) following manufacturer’s instructions. Lactate levels were normalised on total cell number, counted using MultiSizer cell counter (Beckman Coulter).

**Supplemental Figures**

**Supplemental Figure 1.** Schematic representation of ^13^C_6_-glucose (red) and ^13^C_5_-glutamine (blue) incorporation through either the glycolysis end products and/or TCA cycle intermediates. Red and blue colored circles refer to the 13-carbon labeled (^13^C), black circles refers to the 12-carbon unlabeled.

**
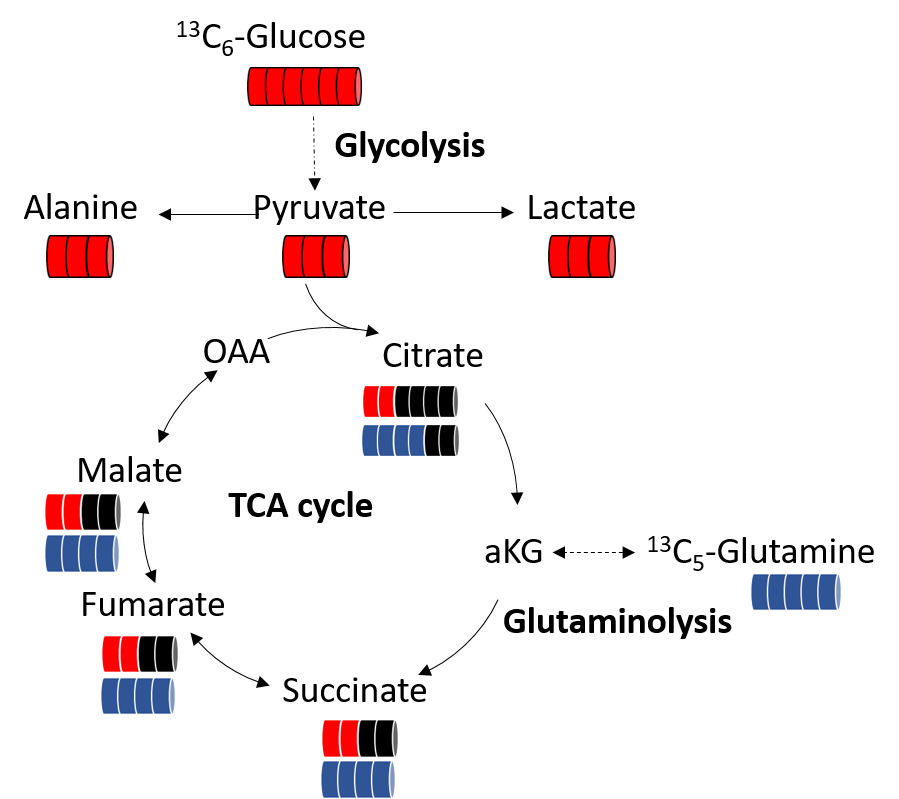
**

**Supplemental Figure 2. A.** Venn diagram of the unique and shared significantly altered proteins (one-way ANOVA and Mann-Whitney-Wilcoxon Test) in NSCLC H1299 cells harbouring K (KRAS^G12C^), S (LKB1^loss^) or KS (KRAS/LKB1) relative to WT (parental background)**. B.** Overrepresented biological processes (PANTHER) of the unique significantly deregulated proteins in presence of KS relative to WT. **C.** Overrepresented biological processes (PANTHER) of the unique significantly deregulated proteins in presence of K relative to WT. **D**. Overrepresented biological processes (PANTHER) of the unique significantly deregulated proteins in presence of S relative to WT.


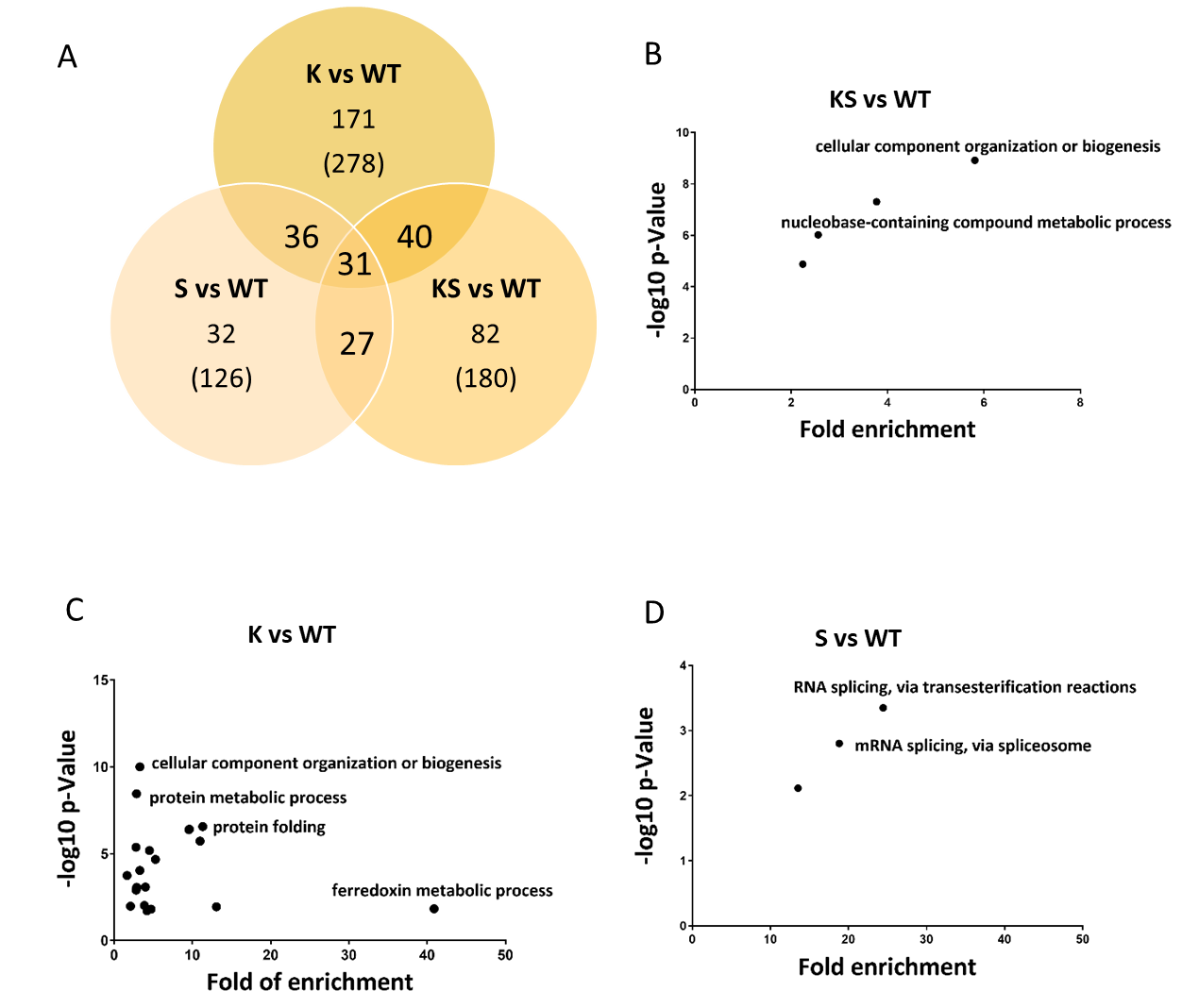


**Supplemental Figure 3. A.** Enriched biochemical pathways in NSCLC NCI-H1299 harbouring KS, K and S relative to WT clone **B.** Enriched biochemical pathways in NSCLC NCI-H1299 harbouring KS relative to single K or S. Tree-maps show the significant biochemical pathways (MetaboAnalyst, v3.0, www.metaboanalyst.ca.). The size of the boxes corresponds to the number of deregulated metabolites (number in the box) mapping into that pathway and the colour the abundance score, computed as reported in Hakimi et al.[8]. Red pathways are characterized by higher average levels of metabolites in the pathway, and dark blue by lower average level.


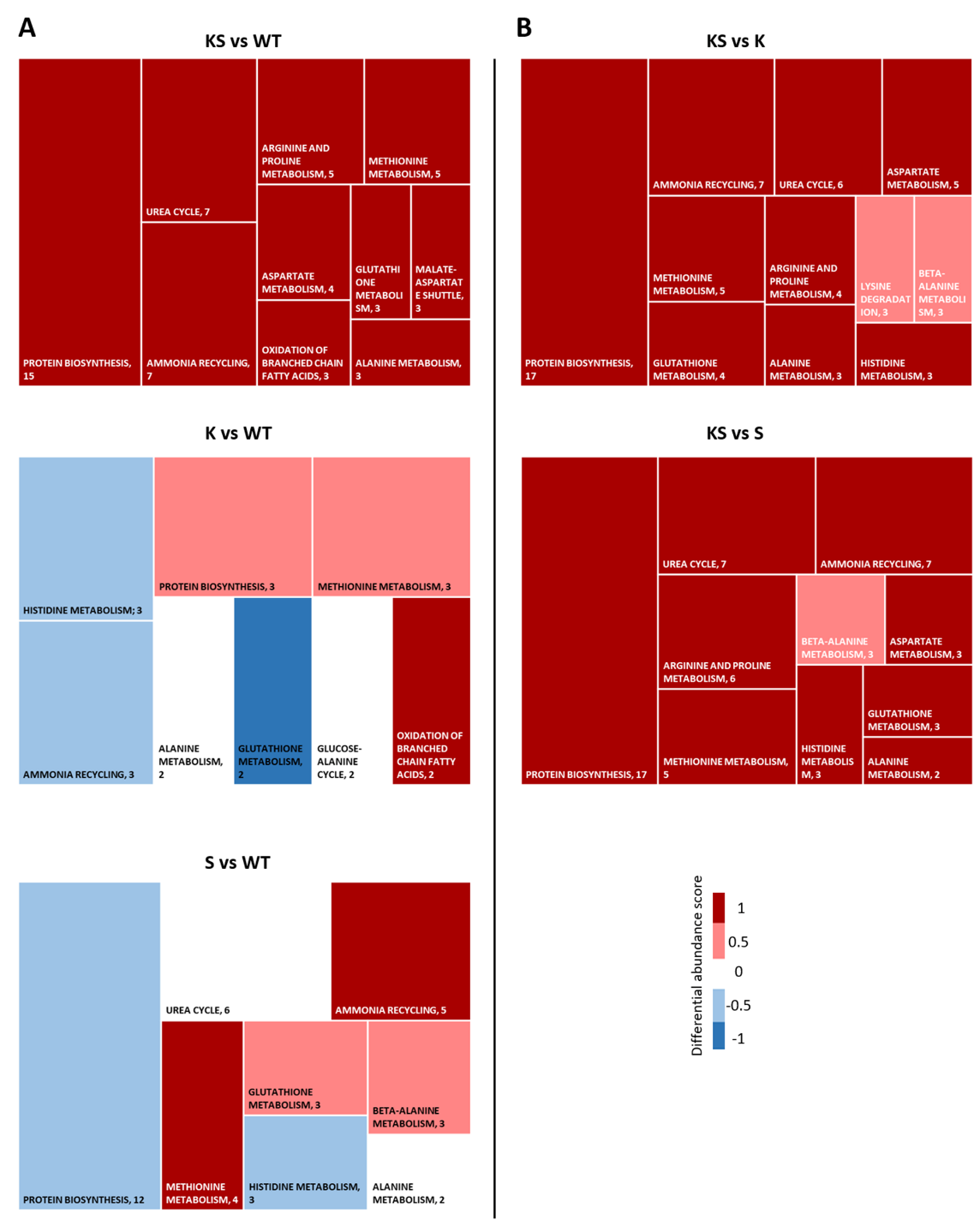


**Supplemental Figure 4.** ^13^C labeling patterns products with ^13^C_6_ glucose as tracer after 20 minute of incubation in NSCLC H1299 KS, K, S and WT clones. M+3 or +6 labelled compounds indicate molecules of those compounds that contain 3 or 6 ^13^C atoms, respectively. Bar graphs indicate mean ± SD, n: three biological replicates. P values were calculated using one-way ANOVA test and Tukey Kramer test post-test for multiple comparisons. * marks significantly differences *p<0.05, **p<0.01.

**
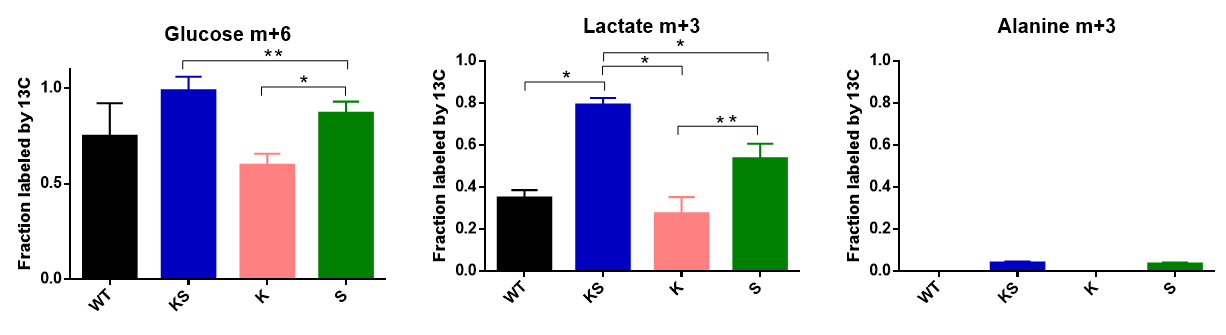
**

**Supplemental Figure 5. A.** Oxygen consumption rate (OCR) changes under mitochondrial stress in each NSCLC NCI-H1299 isogenic clones. We sequentially applied oligomycin, (0.5 mM), FCCP (0.25 mM), and antimycin (1 mM) and rotenone (1 mM), according to the mitochondrial stress kit protocol. **B.** Mitochondrial stress test parameters calculated following manufacture instruction. **C.** Extracellular acidification rare (ECAR) changes under glycolysis stress in each NSCLC NCI-H1299 isogenic clones. We sequentially applied glucose (10mM), olygomycin (1μM) and 2DG (50mM) according to the glycolysis stress test kit protocol. **D.** Glycolysis stress test parameters calculated following manufacture instruction. Bars represent median OCR/ECAR value for six experiments. Error bars represent SEM. Statistical significance was evaluate using two-way ANOVA with Bonferroni's multiple comparisons correction.


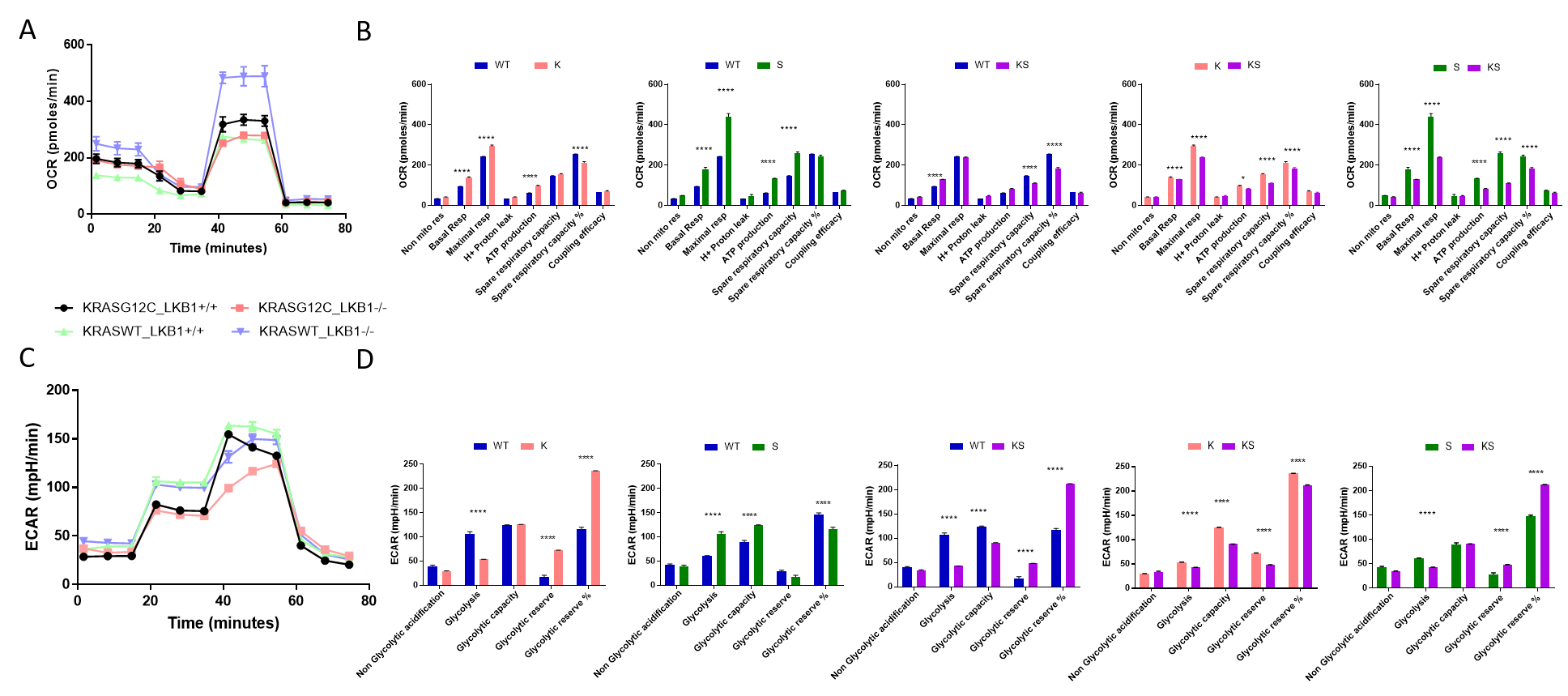


**Supplemental Figure 6.** Growth curve of NSCLC isogenic clones in different culture medium conditions, reported in Figure 5B. Cells were seeded at 30000 cells/mL in 96-well, blank plates and cell growth was analyzed with RealTime-Glo MT Cell Viability Assay. Growth curves were plotted as Normalized LU and represented the mean and SD of three independent replicates.

**
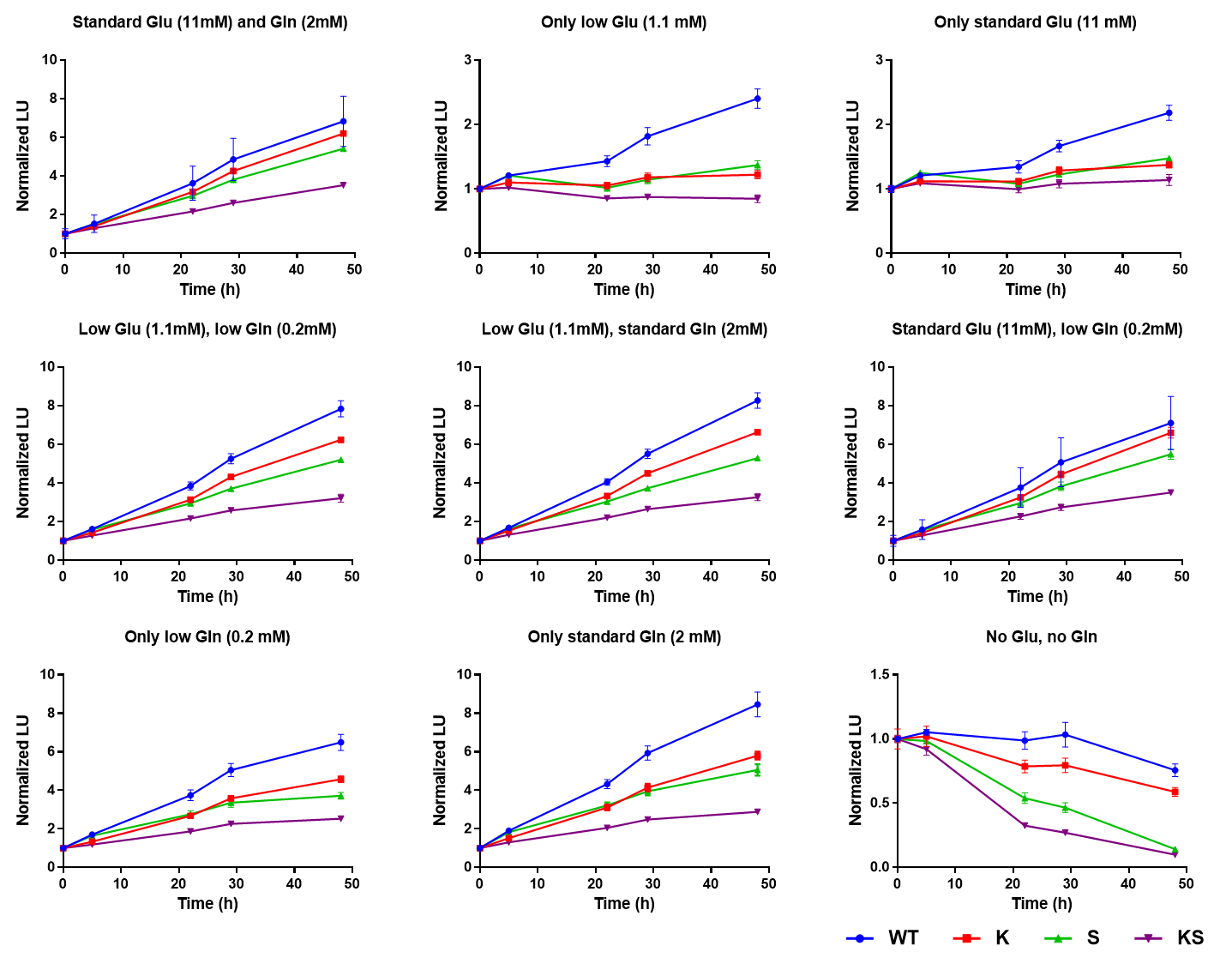
**

**Supplementary References**

1. Cox J, Mann M. MaxQuant enables high peptide identification rates, individualized p.p.b.-range mass accuracies and proteome-wide protein quantification. Nat Biotechnol. 2008;26:1367–72.

2. Cox J, Neuhauser N, Michalski A, Scheltema RA, Olsen JV, Mann M. Andromeda: a peptide search engine integrated into the MaxQuant environment. J Proteome Res. 2011;10:1794–805.

3. Tyanova S, Temu T, Sinitcyn P, Carlson A, Hein MY, Geiger T, et al. The Perseus computational platform for comprehensive analysis of (prote)omics data. Nat Methods. 2016;13:731–40.

4. Mi H, Muruganujan A, Thomas PD. PANTHER in 2013: modeling the evolution of gene function, and other gene attributes, in the context of phylogenetic trees. 2013th ed. an;41;2012 Nov 27.

5. Brunelli L, Caiola E, Marabese M, Broggini M, Pastorelli R. Capturing the metabolomic diversity of KRAS mutants in non-small-cell lung cancer cells. Oncotarget. 2014;5:4722–31.

6. Fuhrer T, Heer D, Begemann B, Zamboni N. High-throughput, accurate mass metabolome profiling of cellular extracts by flow injection-time-of-flight mass spectrometry. Anal Chem. 2011;83:7074–80.

7. Brunelli L, Caiola E, Marabese M, Broggini M, Pastorelli R. Comparative metabolomics profiling of isogenic KRAS wild type and mutant NSCLC cells in vitro and in vivo. Sci Rep. 2016;6:28398.

8. Hakimi AA, Reznik E, Lee C-H, Creighton CJ, Brannon AR, Luna A, et al. An Integrated Metabolic Atlas of Clear Cell Renal Cell Carcinoma. Cancer Cell. 2016;29:104–16.
